# Engineering CRISPR interference system in *Klebsiella pneumoniae* for attenuating lactic acid synthesis

**DOI:** 10.1186/s12934-018-0903-1

**Published:** 2018-04-05

**Authors:** Jingxuan Wang, Peng Zhao, Ying Li, Lida Xu, Pingfang Tian

**Affiliations:** 1 Beijing Key Laboratory of Bioprocess, College of Life Science and Technology, Beijing University of Chemical Technology, Beijing, 100029 People’s Republic of China; 2 College of Biochemical Engineering, Beijing Union University, Beijing, 100023 People’s Republic of China

**Keywords:** CRISPR interference, *Klebsiella pneumoniae*, 3-Hydroxypropionic acid, Lactic acid, Lactate dehydrogenase

## Abstract

**Background:**

*Klebsiella pneumoniae* is a promising industrial species for bioproduction of bulk chemicals such as 1,3-propanediol, 2,3-butanediol and 3-hydroxypropionic acid (3-HP). However, lactic acid is a troublesome by-product when optimizing for 3-HP production. Therefore, it is highly desirable to minimize lactic acid.

**Results:**

Here, we show that lactic acid synthesis can be largely blocked by an engineered CRISPR interference (CRISPRi) system in *K*. *pneumoniae*. EGFP was recruited as a reporter of this CRISPRi system. Fluorescence assay of this CRISPRi system showed that enhanced green fluorescent protein (EGFP) expression level was repressed by 85–90%. To further test this CRISPRi system, guide RNAs were designed to individually or simultaneously target four lactate-producing enzyme genes. Results showed that all lactate-producing enzyme genes were significantly repressed. Notably, d-lactate dehydrogenase (ldhA) was shown to be the most influential enzyme for lactic acid formation in micro-aerobic conditions, as inhibiting *ldhA* alone led to lactic acid level similar to simultaneously repressing four genes. In shake flask cultivation, the strain coexpressing *puuC* (an aldehyde dehydrogenase catalyzing 3-hydroxypropionaldehyde to 3-HP) and dCas9-sgRNA inhibiting *ldhA* produced 1.37-fold 3-HP relative to the reference strain. Furthermore, in bioreactor cultivation, this CRISPRi strain inhibiting *ldhA* produced 36.7 g/L 3-HP, but only generated 1 g/L lactic acid. Clearly, this engineered CRISPRi system largely simplified downstream separation of 3-HP from its isomer lactic acid, an extreme challenge for 3-HP bioprocess.

**Conclusions:**

This study offers a deep understanding of lactic acid metabolism in diverse species, and we believe that this CRISPRi system will facilitate biomanufacturing and functional genome studies of *K*. *pneumoniae* or beyond.

**Electronic supplementary material:**

The online version of this article (10.1186/s12934-018-0903-1) contains supplementary material, which is available to authorized users.

## Background

**Fig. 1:**
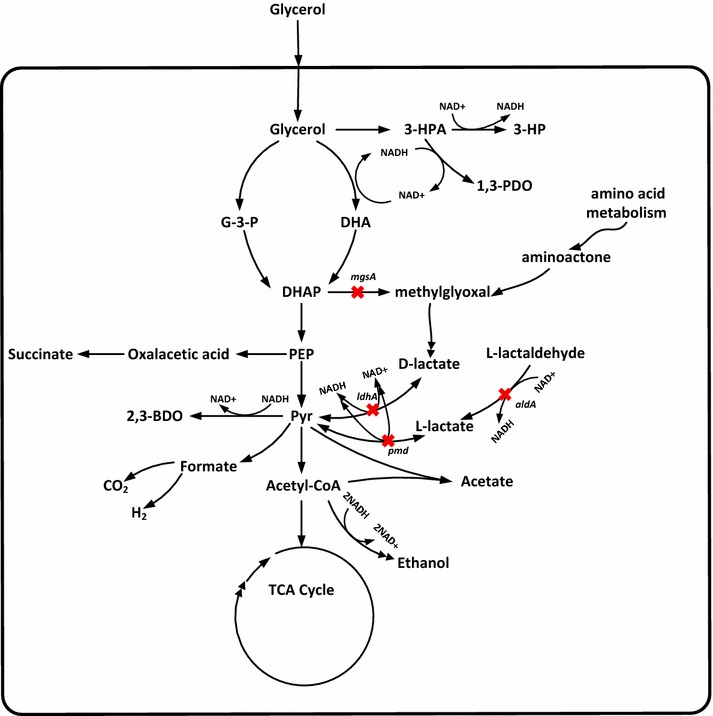
Glycerol and lactic acid pathways in *Klebsiella pneumoniae*. 3-HPA, 3-hydroxypropionaldehyde; 3-HP, 3-hydroxypropionic acid; 1,3-PDO, 1,3-propanediol; G-3-P, sn-glycerol 3-phosphate; DHA, dihydroxyacetone; DHAP, dihydroxyacetone phosphate; PEP, phosphoenolpyruvate; Pyr, pyruvate; 2,3-BDO, 2,3-butanediol; *ldhA*, d-lactate dehydrogenase gene; *pmd*, l-lactate dehydrogenase gene; *mgsA*, methylglyoxal synthase gene; *aldA*, lactaldehyde dehydrogenase gene. Red cross indicates enzyme genes to be repressed by CRISPRi system

For bioproduction of chemicals, byproducts are problematic because they not only consume cellular resources but also entangle downstream separation. Conventional genetic engineering strategies to attenuate byproducts formation mainly rely on deletion or repression of their biosynthesis genes [1–4]. However, these approaches in most cases compromise cell growth which in turn hampers the production of desired metabolites [5]. In recent years, *Klebsiella pneumoniae* has attracted much attention because it can naturally convert glycerol to a range of economically important bulk chemicals including 1,3-propanediol (1,3-PDO), 2,3-butanediol (2,3-BDO), 3-hydroxypropionic acid (3-HP) and d-lactic acid [1, 2, 6–11]. Glycerol metabolism in *K*. *pneumoniae* is mediated by the *dha* and *glp* regulons in anaerobic or micro-aerobic conditions [6, 9, 12]. The *dha* regulon involves glycerol reduction and oxidation pathways (Fig. 1). In the reduction pathways, glycerol is converted to 3-hydroxypropionaldehyde (3-HPA) by GDHt (encoded by *dhaB* cluster, GenBank No. U30903). Next, 3-HPA is converted to 1,3-PDO by 1,3-propanediol dehydrogenase (PDOR, encoded by *dhaT*) [13]. 3-HPA is also converted to 3-HP by aldehyde dehydrogenase (ALDH) with NAD^+^ as a cofactor [9]. In the glycerol oxidation pathways, a series of metabolites such as ATP, NAD^+^, 2,3-BDO and lactic acid are generated to sustain cell growth and benefit 1,3-PDO and 3-HP production [9]. Of various metabolites, lactic acid is the major byproduct in the production of 1,3-PDO, 2,3-BDO and 3-HP [14]. The formation of lactic acid not only consumes carbon source but also entangles downstream separation [2]. It is extremely challenging to separate lactic acid (2-hydroxy propionic acid) from 3-HP (3-hydroxy propionic acid) because they are isomers. On the other hand, although the formation of lactic acid consumes carbon flux, it also augments carbon flux toward glycerol reduction pathways. Especially, when dissolved oxygen is insufficient, lactic acid synthesis leads to NAD^+^ regeneration which in return drives 3-HP production because NAD^+^ is the cofactor of aldehyde dehydrogenase that catalyzes 3-HPA to 3-HP [7, 9]. In other words, 3-HP production consumes NAD^+^, and the lack of NAD^+^ in turn necessitates lactic acid synthesis. Clearly, there is interdependence between lactic acid formation and 3-HP production. Hence, knockdown instead of deletion of lactic acid pathways is beneficial for 3-HP production.

CRISPR technology opens avenue for simultaneous knockdown or knockout of multiple genes due to an array of single guide RNAs (sgRNAs) that direct dCas9 or Cas9 to interference or edit target genes [15–19]. The dCas9-sgRNA-based CRISPR interference (CRISPRi) tools mainly include CRISPR activation and CRISPR repression [20, 21]. The dCas9-sgRNA complex activates gene expression when dCas9 is fused with the omega subunit of RNA polymerase, while it represses gene expression when dCas9 binds a promoter or an open reading frame (ORF) [21]. The Cas9-based DNA cleavage largely relies on DNA repair mechanisms including homologous recombination in prokaryotes and non-homologous end joining (NHEJ) in eukaryotes [17]. Unlike Cas9-based DNA editing, CRISPRi is independent of DNA repair and thus can be easily applied in microbes lacking the NHEJ pathway or for which no efficient homologous recombination approach is available. Furthermore, compared with CRISPR editing that may lead to slowed cell growth or even cell death, CRISPRi could be more appropriate for modulating multiple genes.

In view of above information, we anticipated that CRISPRi may be ideal for modulating lactic acid metabolism which is subjected to multiple factors. To validate this prediction, in this study we developed CRISPRi system in *K. pneumoniae*. Detailed analysis of glycerol consumption, cell growth, metabolites formation and gene transcription was to systematically assess the performance of CRISPRi system in *K. pneumoniae*. Shake-flask and bioreactor cultivation of the recombinant *K. pneumoniae* strain harboring CRISPRi vectors (hereafter refers to as CRISPRi strain) were to determine the key enzymes affecting lactic acid synthesis. Overall, this study was to exploit CRISPRi system for basic research and metabolic engineering of *K. pneumoniae*.

## Results

### Fluorescence assay of dCas9-gRNA-mediated transcription repression in *K. pneumoniae*

**Fig. 2:**
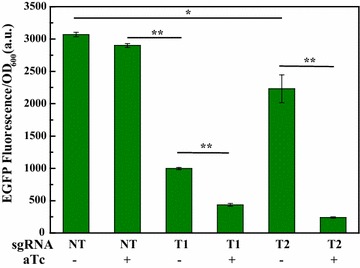
Fluorescence assay of the engineered CRISPRi system in *K*. *pneumoniae* using EGFP as a reporter. NT, *K*. *pneumoniae* strain harboring CRISPRi vector without guide RNA; T_1_ and T_2_, *K*. *pneumoniae* strains harboring CRISPRi vectors targeting the different regions of promoter upstream of EGFP gene; aTc, anhydrotetracycline; “+”, aTc induction; “−”, without aTc. *P < 0.05; **P < 0.01

The dCas9 was derived from *Streptococcus pyogenes*, and CRISPRi system was developed to dissect lactic acid metabolism. To determine whether it functioned in *K*. *pneumoniae*, EGFP was employed as a reporter under *tac* promoter. To ensure CRISPRi efficiency, two candidate guide RNAs T_1_ and T_2_ toward the different regions of *tac* promoter were designed and chemically synthesized. The CRISPRi vector with non-targeting sgRNA was used as a control. The fluorescence intensity of single strain was calculated as the total fluorescence intensity divided by OD_600_ value. Results showed that EGFP expression was significantly down-regulated by CRISPRi system (Fig. 2). Compared with the control strain NT with non-targeting guide RNA, strains T1 and T2 showed a remarkable decrease in the fluorescence intensity even if anhydrotetracycline (aTc) was absent. Strains T_1_ and T_2_ respectively exhibited 65 and 23% inhibition on EGFP when aTc was absent. Notably, T_1_ and T_2_ respectively displayed 85 and 90% inhibition on EGFP level when aTc was added into medium. Overall these results indicated that CRISPRi system significantly repressed EGFP expression, although *tet* promoter failed to tightly control dCas9 expression in *K*. *pneumoniae*. Namely, there existed leaky expression of dCas9.

### Repression of lactate-producing enzyme genes by CRISPRi system

**Fig. 3:**
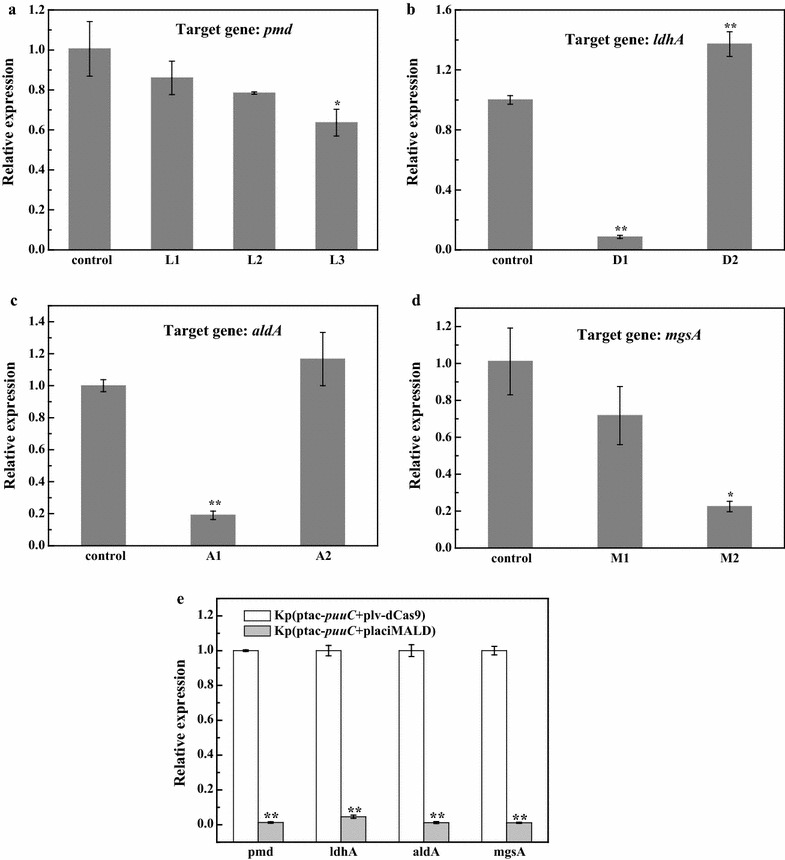
Relative expression levels of lactate-producing enzyme genes in *K*. *pneumoniae* harboring CRISPRi system. **a** Relative expression level of *pmd* gene. L1, L2 and L3 indicate three candidate guide RNAs targeting *pmd* gene. **b** Relative expression level of *ldhA* gene. D1 and D2 indicate two candidate guide RNAs targeting *ldhA* gene. **c** Relative expression level of *aldA* gene. A1 and A2 stand for two candidate guide RNAs targeting *aldA* gene. **d** Relative expression level of *mgsA* gene. M1 and M2 refer to two candidate guide RNAs targeting *mgsA* gene. Control, without guide RNA. **e** Relative expression levels of *pmd*, *ldhA*, *aldA* and *mgsA* genes in Kp(ptac-*puuC *+ placiMALD) at 12 h. Kp(ptac-*puuC *+ plv-dCas9), recombinant *K*. *pneumoniae* strain harboring vector ptac-*puuC* and vector plv-dCas9 where Cas9 was inactive; Kp(ptac-*puuC *+ placiMALD), recombinant *K*. *pneumoniae* strain harboring vector ptac-*puuC* and CRISPRi vector placiMALD simultaneously targeting four lactate-producing enzyme genes *pmd*, *ldhA*, *aldA* and *mgsA.* *P < 0.05; **P < 0.01

To further validate the CRISPRi system, it was harnessed to repress lactate-producing enzyme genes. In *K. pneumoniae*, many enzymes potentially contribute to lactic acid synthesis, including l-lactate dehydrogenase (*pmd*), d-lactate dehydrogenase (*ldhA*), lactaldehyde dehydrogenase (*aldA*) and methylglyoxal (*mgsA*). To determine the key enzymes affecting lactic acid formation, an array of sgRNAs targeting enzyme genes were designed and chemically synthesized. For each lactate-producing enzyme gene, two or three candidate sgRNAs were subjected to screening. The sgRNAs were designed by using online software CRISPR direct (http://crispr.dbcls.jp/doc/) [22]. In principle, sgRNA sequence should target transcription initiation site especially that in non-template strand [23]. In addition, sgRNA sequence was used as a query to search against *K*. *pneumoniae* genome to avoid targeting homologous sequence and off-target. The secondary structure of sgRNAs were predicted by using online Quikfold algorithm from the UNAFold package [24]. All engineered vectors were confirmed by sequencing. The CRISPRi vectors targeting different lactate-producing enzyme genes were transformed into the previously engineered 3-HP-producing strain Kp(ptac-*puuC*) [2], resulting in recombinant strain Kp(ptac-*puuC *+ placiL), Kp(ptac-*puuC *+ placiD), Kp(ptac-*puuC *+ placiA) and Kp(ptac-*puuC *+ placiM). Next, quantitative real-time PCR (qRT-PCR) was performed to examine CRISPRi efficiency toward lactate-producing enzyme genes. Results showed that guide RNAs L1 and L2 failed to significantly inhibit *pmd* gene (Fig. 3a). However, for three CRISPRi strains targeting *ldhA*, *aldA* and *mgsA*, at least one sgRNA showed inhibition activity (Fig. 3b–d). To simultaneously repress four lactate-producing enzyme genes, four well-functioned sgRNAs (L3 for *pmd*, D1 for *ldhA*, A2 for *aldA*, M2 for *mgsA*) were joined together to form a new vector named placiMALD. To investigate the performance of CRISPRi system, we engineered a vector named Kp(ptac-*puuC *+ placiMALD), where vectors dCas9-sgRNA and puuC were coexpressed. The qRT-PCR assay showed that CRISPRi vector placiMALD significantly repressed all lactate-producing enzyme genes (Fig. 3e).

### Shake flask cultivation of the CRISPRi strains

**Fig. 4:**
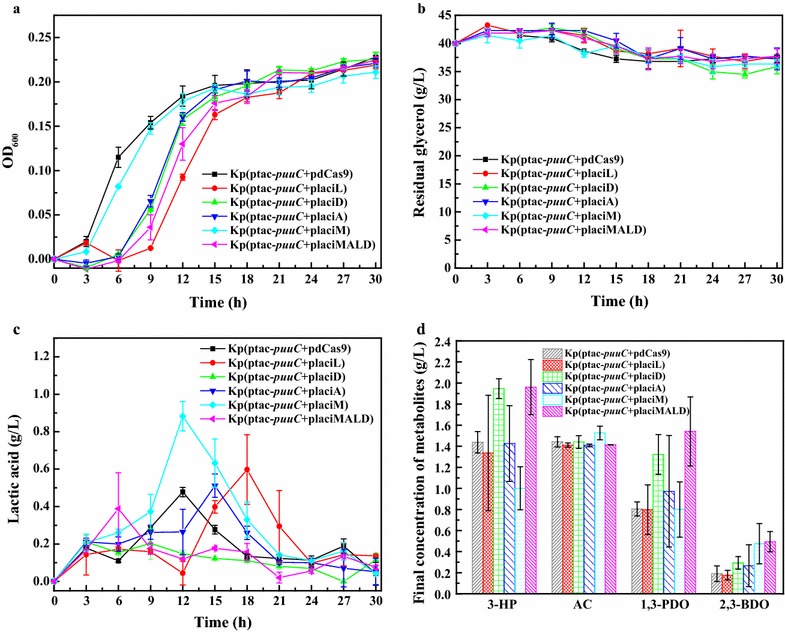
Shake-flask cultivation of *K*. *pneumoniae* strain harboring CRISPRi system. **a** Growth curve of the recombinant *K. pneumoniae* strain harboring CRISPRi vector and aldehyde dehydrogenase puuC expression vector. **b** Residual glycerol concentration. **c** Lactic acid concentration. **d** Metabolites concentration after 30 h cultivation. 3-HP, 3-hydroxypropionic acid; AC, acetic acid; 1,3-PDO, 1,3-propanediol; 2,3-BDO, 2,3-butanediol. placiL, CRISPRi vector targeting l-lactate dehydrogenase gene *pmb*; placiD, CRISPRi vector targeting d-lactate dehydrogenase gene *ldhA*; placiA, CRISPRi vector targeting lactaldehyde dehydrogenase gene *aldA*; placiM, CRISPRi vector targeting methylglyoxal synthase gene *mgsA*; placiMALD, CRISPRi vector targeting all above four genes. Kp(ptac-puuC + pdCas9), recombinant *K. pneumoniae* strain harboring *tac* promoter-driven puuC expression vector and dCas9 expression vector; Kp(ptac-puuC + placiL), recombinant *K. pneumoniae* strain harboring puuC expression vector and CRISPRi vector targeting l-lactate dehydrogenase gene *pmb*; Kp(ptac-puuC + placiD), recombinant *K. pneumoniae* strain harboring puuC expression vector and CRISPRi vector targeting d-lactate dehydrogenase gene *ldhA*; Kp(ptac-puuC + placiA), recombinant *K. pneumoniae* strain harboring puuC expression vector and CRISPRi vector targeting lactaldehyde dehydrogenase gene *alhA*; Kp(ptac-puuC + placiM), recombinant *K. pneumoniae* strain carrying puuC expression vector and CRISPRi vector targeting methylglyoxal synthase gene *mgsA*; Kp(ptac-puuC + placiMALD), recombinant *K. pneumoniae* strain harboring puuC expression vector and CRISPRi vector simultaneously targeting four genes *mgsA*, *aldA*, *pmb* and *ldhA*

To overproduce 3-HP and attenuate lactic acid formation, PuuC was coexpressed with dCas9. To do so, we engineered five recombinant strains designated Kp(ptac-*puuC *+ placiL), Kp(ptac-*puuC *+ placiD), Kp(ptac-*puuC *+ placiA), Kp(ptac-*puuC *+ placiM) and Kp(ptac-*puuC *+ placiMALD). These strains were fermented in micro-aerobic conditions with Kp(ptac-*puuC *+ pdCas9) as the control. In 15 h cultivation, these strains showed retarded growth and postponed log phase with exception of the strain Kp(ptac-*puuC *+ placiM), where “placiM” indicates CRISPRi vector targeting *mgsA* gene (Fig. 4a). Interestingly, in stationary phase, all these strains demonstrated similar OD_600_ value with the control Kp(ptac-*puuC *+ pdCas9). However, due to harboring of two vectors pSg and ptac-*puuC *+ Cas9, glycerol consumption was largely retarded (Fig. 4b). For lactic acid formation, the strain harboring CRISPRi vector targeting *mgsA* had highest lactic acid peak (Fig. 4c). In contrast, the strains Kp(ptac-*puuC *+ placiD) and Kp(ptac-*puuC *+ placiMALD) produced less lactic acid (Fig. 4c). For other strains, lactic acid accumulated in log phase but declined in stationary phase. In addition, strains Kp(ptac-*puuC *+ placiD) and Kp(ptac-*puuC *+ placiMALD) produced more 3-HP and 1,3-PDO at 30 h than other strains did. Only the strain Kp(ptac-*puuC *+ placiM) produced less 3-HP than the control. For acetic acid and 2,3-BDO production, no significant difference was observed in all strains (Fig. 4d).

### Fed-batch cultivation of CRISPRi strains

**Fig. 5:**
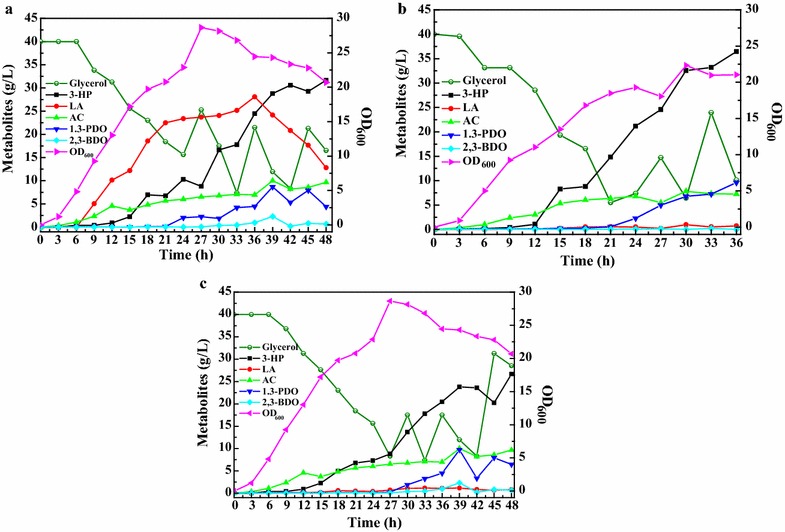
Bioreactor cultivation of the *K. pneumoniae* strains harboring CRISPRi system. **a** Kp(ptac-puuC + pdCas9), recombinant *K. pneumoniae* strain harboring aldehyde dehydrogenase puuC expression vector and dCas9 expression vector; **b** Kp(ptac-puuC + placiD), recombinant *K. pneumoniae* strain harboring aldehyde dehydrogenase puuC expression vector and CRISPRi vector targeting d-lactate dehydrogenase gene *ldhA*; **c** Kp(ptac-puuC + placiMALD), recombinant *K. pneumoniae* strain harboring puuC expression vector and CRISPRi vector targeting four lactate-producing enzyme genes *mgsA*, *aldA*, *pmb* and *ldhA*. 3-HP, 3-hydroxypropionic acid; LA, lactic acid; AC, acetic acid; 1,3-PDO, 1,3-propanediol; 2, 3-BDO, 2,3-butanediol

As mentioned, the strains Kp(ptac-*puuC *+ placiD) and Kp(ptac-*puuC *+ placiMALD) produced less lactic acid in shake flasks relative to other strains (Fig. 4c). To further investigate the performance of CRISPRi system, the above two strains were independently cultivated in 5 L bioreactor. Results showed that only trace amount of lactic acid was generated during entire fermentation process (Fig. 5b, c). In addition, due to PuuC overexpression, the strain Kp(ptac-*puuC *+ placiD) generated 36.7 g/L 3-HP at 36 h (Fig. 5b), with 41.7% of glycerol conversion ratio (GCR) and 1.02 g/L/h of productivity (Table 1). The GCR is calculated as the each metabolite concentration divided by the total glycerol concentration. We also found that the strain Kp(ptac-*puuC *+ placiMALD) harboring CRISPRi vector targeting all four lactate-producing enzyme genes produced 26.9 g/L 3-HP at 48 h (Fig. 5c), with GCR of 34.6% and 0.56 g/L/h of productivity (Table 2). Compared with Kp(ptac-*puuC *+ placiD), the strain Kp(ptac-*puuC *+ placiMALD) produced more acetic acid to compensate cofactor for cell growth. For above two strains, the overall conversion ratio from glycerol to major metabolites was around 60–70%, indicating that partial carbon source flowed into other pathways. It should be pointed out that CRISPRi system imposed a burden on cell growth. This finding is consistent with other study [25]. As Fig. 5 shown, the highest OD_600_ was only 20–30.

**Table 1:** Carbon distribution of Kp(ptac-*puuC *+ placiD) in a 5 L bioreactor (36 h)

Metabolites	3-HP	LA	AA	1, 3-PDO	2, 3-BDO	Total
Titer (mM)	407	7	123	126	0	976
Titer (g/L)	36.7	0.7	7.4	9.6	0	54.4
GCR (%)	41.7	0.7	12.6	12.9	0	67.9

*3-HP* 3-hydroxypropionic acid, *LA* lactic acid, *AA* acetic acid, *1,3-PDO* 1,3-propanediol, *2,3-BDO* 2,3-butanediol, *GCR* glycerol conversion ratio

Kp(ptac-*puuC *+ placiD), recombinant *K. pneumoniae* strain harboring vector overexpressing puuC and CRISPRi vector targeting *ldhA* gene

**Table 2:** Carbon distribution of Kp(ptac-*puuC *+ placiMALD) in a 5 L bioreactor (48 h)

Metabolites	3-HP	LA	AA	1,3-PDO	2,3-BDO	Total
Titer (mM)	298	7	163	84	7	861
Titer (g/L)	26.9	0.7	9.8	6.4	0.7	44.5
GCR (%)	34.6	0.8	18.9	9.8	0.8	64.9

*3-HP* 3-hydroxypropionic acid, *LA* lactic acid, *AA* acetic acid, *1,3-PDO* 1,3-propanediol, *2,3-BDO* 2,3-butanediol, *GCR* glycerol conversion ratio

Kp(ptac-*puuC *+ placiD), recombinant *K. pneumoniae* harboring vector overexpressing puuC and CRISPRi vector simultaneously targeting four lactate-producing enzyme genes *ldhA*, *aldA*, *pmd* and *mgsA*

### Dissecting interactions of lactate-producing enzyme genes by CRISPRi strains

**Fig. 6:**
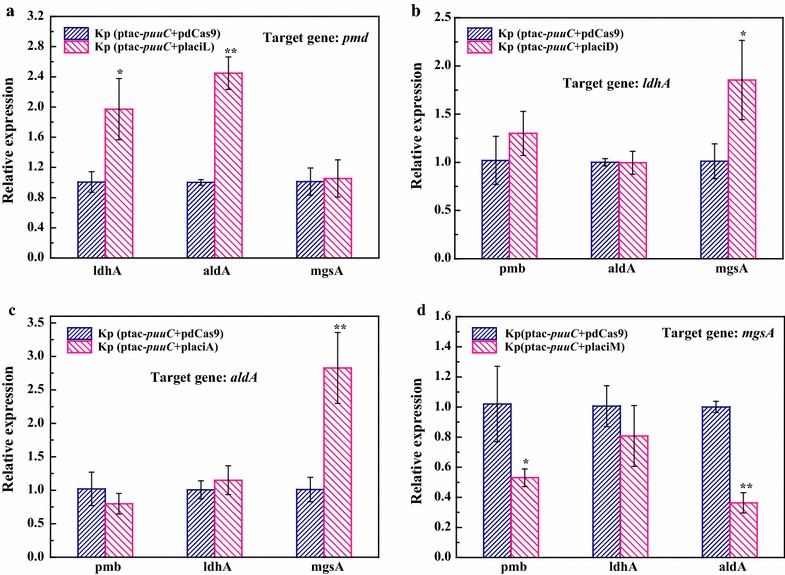
Relative expression levels of lactate-producing enzyme genes in *K. pneumoniae* strain harboring CRISPRi system. **a** Relative expression levels of *ldhA*, *aldA* and *mgsA* genes in *K. pneumoniae* strain harboring CRISPRi system targeting *pmd* gene; **b** Relative expression levels of *pmd*, *aldA* and *mgsA* genes in *K. pneumoniae* strain harboring CRISPRi system targeting *ldhA*; **c** Relative expression levels of *pmd*, *ldhA* and *mgsA* genes in *K. pneumoniae* strain harboring CRISPRi system targeting *aldA*; **d** Relative expression levels of *pmd*, *ldhA* and *aldA* genes in *K. pneumoniae* strain harboring CRISPRi system targeting *mgsA*. Kp(ptac-*puuC *+ pdCas9), recombinant *K. pneumoniae* strain harboring aldehyde dehydrogenase puuC expression vector and dCas9 expression vector; Kp(ptac-*puuC *+ placiL), recombinant *K. pneumoniae* strain harboring puuC expression vector and CRISPRi vector targeting l-lactate dehydrogenase gene *pmb*; Kp(ptac-*puuC *+ placiD), recombinant *K. pneumoniae* strain harboring puuC expression vector and CRISPRi vector targeting d-lactate dehydrogenase gene *ldhA*; Kp(ptac-*puuC *+ placiA), recombinant *K. pneumoniae* strain harboring puuC expression vector and CRISPRi vector targeting lactaldehyde dehydrogenase gene *aldA*; Kp(ptac-*puuC *+ placiM), recombinant *K. pneumoniae* strain harboring puuC expression vector and CRISPRi vector targeting methylglyoxal synthase gene *mgsA.* *P < 0.05; **P < 0.01

A number of enzymes potentially contribute to lactic acid synthesis (Additional file 1: Fig. S1). To decipher their interactions, a total of four CRISPRi strains targeting different lactic acid-producing enzyme genes were subjected to qRT-PCR analysis. Results showed that repressing anyone of lactic acid-producing enzyme genes led to up-regulation or down-regulation of other enzyme genes (Fig. 6). For example, in strain Kp(ptac-*puuC *+ placiL) targeting *pmd* gene, both *ldhA* and *aldA* genes were upregulated, while *mgsA* gene was less affected (Fig. 6a). By contrast, the *mgsA* gene was significantly upregulated in both Kp(ptac-*puuC *+ placiD) and Kp(ptac-*puuC *+ placiA) (Fig. 6b, c), where *ldhA* and *aldA* genes were respectively targeted by CRISPRi. In strain Kp(ptac-*puuC *+ placiM) targeting *mgsA*, both *pmb* and *aldA* genes were downregulated (Fig. 6d).

## Discussion

In this work, we developed CRISPRi system in *K*. *pneumoniae* to attenuate lactic acid formation. The dCas9 was derived from *S*. *pyogenes*, and considering the performance of CRISPRi system is largely dependent on guide RNA, we designed several candidate guide RNAs and screened the best. The qRT-PCR results showed that all four lactate-producing enzyme genes were transcriptionally repressed (Figs. 3e, 6). In micro-aerobic conditions, the strain Kp(ptac-puuC + placiD) coexpressing PuuC and *ldhA*-targeting dCas9-sgRNA complex produced similar level of lactic acid with the strain Kp(ptac-puuC + placiMALD) coexpressing puuC and dCas9-sgRNA targeting four lactate-producing enzyme genes (Figs. 4c, 5b, c), indicating that *ldhA* is the predominant gene for lactic acid synthesis. Importantly, in fed-batch cultivation, although all CRISPRi strains produced similar levels of lactic acid at 24–30 h (Fig. 4c), however the strains Kp(ptac-puuC + placiD) and Kp(ptac-puuC + placiMALD) generated low levels of lactic acid during entire fermentation process. Clearly, this offers flexibility for fermentation, because lactic acid maintains minimal throughout (Fig. 5b, c). It should be pointed out that leaky expression of dCas9 occurred. This could be partially ascribed to unknown aTc analogs in yeast extract or possible influences of TetR/tetO on aTc. This engineered CRISPRi system needs amelioration prior to real-world application.

Our previous study reported the high titer of 3-HP (83.8 g/L) in *K*. *pneumoniae* (2), which is higher than CRISPRi strains. The low 3-HP production in CRISPRi strains could be partially attributed to the expression of dCas9 which imposed a heavy burden on cells. Despite low 3-HP production, lactic acid was attenuated by the engineered CRISPRi system, which simplified downstream separation. The remaining lactic acid in bioreactor is limited, and can be converted to other metabolites by expressing an enzyme. Alternatively, lactic acid can be separated using preparative chromatography. Clearly, this is uneconomical for large-scale fermentation. If 3-HP is converted to acrylic acid simply by high temperature, the remaining lactic acid may not affect this process. In fact, separation of 3-HP from lactic acid is extremely challenging because they are isomers. So far, nearly no techniques can efficiently separate 3-HP from lactic acid. Lactic acid is mainly generated from pyruvate and its synthesis pathways are conserved in nearly all organisms (Additional file 1: Fig. S1). Conventional approaches to block lactic acid formation rely mainly on the deletion of enzyme genes or optimization of fermentation conditions [2, 7]. However, these approaches neglect the complexity of lactic acid pathways and their contribution to the production of desired metabolites such as 3-HP and 1,3-PDO [9]. From the viewpoint of evolution, the complexity of lactic acid pathways enable bacteria to buffer external stimuli and adapt harsh environment. Deduced from Additional file 1: Fig. S1, deletion of one or two lactic acid pathways might not be lethal to *K*. *pneumoniae* owing to tailored compensation mechanism of lactic acid synthesis. That is, repression rather than deletion of lactic acid-synthesizing enzyme genes is appropriate for metabolic engineering purposes. As such, CRISPRi was exploited to decipher and tune lactic acid metabolism.

In this study, the *ldhA* gene was shown to be pivotal for lactic acid accumulation when glycerol was the sole carbon source and micro-aerobic conditions were maintained. As shown in Figs. 4c, 5b, inhibiting *ldhA* gene alone almost completely blocked lactic acid synthesis. This may be explained by the following reasons: (i) 3-HP production relies on PuuC overexpression which consumes NAD^+^ and ATP [2]. Since lactic acid biosynthesis is accompanied by the generation of NAD^+^ and ATP, lactic acid was thus synthesized. (ii) Although l-lactate dehydrogenase (*pmb*) and lactaldehyde dehydrogenase (*aldA*) catalyze the formation of l-lactic acid (Fig. 1, Additional file 1: Fig. S1), however, the corresponding CRISPRi strains produced more lactic acid compared with the CRISPRi strain targeting d-lactate-producing enzyme gene (Fig. 4c). Presumably, d-lactate dehydrogenase contributed largely to lactic acid level. (iii) It has been reported that lactaldehyde can be synthesized through fucose and rhamnose metabolisms [26] (Additional file 1: Fig. S1) and lactaldehyde may serve as an intermediate to alleviate the cytotoxicity of methylglyoxal. However, CRISPRi-based inactivation of lactaldehyde dehydrogenase (*aldA*) and methylglyoxal synthase (*mgsA*) failed to effectively block lactic acid production (Fig. 4c), indicating that lactic acid biosynthesis through methylglyoxal and lactaldehyde pathways may be minimal in current conditions, and methylglyoxal detoxification has little impacts on lactate production and cell growth. Another finding of this study is carbon flux compensation between lactate-producing pathways, as repressing one pathway elicited other pathways (Fig. 6).

CRISPRi can simultaneously repress multiple genes due to recruitment of guide RNAs ([18, 25, 27], Fig. 4c). For enzymes in central pathways, deleting their synthesis genes may impede cell growth or even elicit cell death [5]. CRISPRi is an ideal tool to coordinate cell growth and bioproduction of desired metabolites. Furthermore, unlike RNA interference which represses gene expression at posttranscriptional level, CRISPRi suppresses gene expression at transcriptional level and thus consumes less cellular resources. More importantly, CRISPRi can be easily applied in microbes lacking NHEJ pathway or for which no efficient homologous recombination approach is available. Hence, we believe that the CRISRPi system developed in this study will facilitate functional characterization of key genes and metabolic engineering of *K*. *pneumoniae*.

## Methods

### Strains, medium and chemicals

Strains of *K. pneumoniae* DSM 2026 and *E. coli* Top10 were purchased from DSMZ GmbH, Germany. Strain *K. pneumoniae* DSM 2026 was used as the host for development of CRISPRi system. *E*. *coli* Top10 was used for gene cloning (Additional file 2: Fig. S2, Additional file 3: Table S1). In vector construction experiments, all strains were grown in LB medium containing the following components per liter: 5 g yeast extract, 10 g NaCl, 10 g peptone, and 50 mg kanamycin or 34 mg chloramphenicol. In CRISPRi experiments, strains were grown in fermentation medium containing the following components per liter: K_2_HPO_4_·3H_2_O, 3.4 g; KH_2_PO_4_, 1.3 g; (NH_4_)_2_SO_4_, 4 g; MgSO_4_·7H_2_O, 0.5 g; CaCO_3_, 0.1 g; yeast extract, 3 g; glycerol, 40 g; and 1.25 mL of trace element solution. The trace element solution contained the following components per liter: FeSO_4_, 32 g; ZnCl_2_·6H_2_O, 2.72 g; MnCl_2_·4H_2_O, 0.68 g; CoCl_2_·6H_2_O, 1.88 g; H_3_BO_3_, 0.24 g; Na_2_MoO_4_, 0.02 g; CuCl_2_·2H_2_O, 1.88 g and 40 mL concentrated HCl. Restriction enzymes, taq DNA polymerase and T4 DNA ligase were purchased from TaKaRa (Dalian, China). Primer synthesis and DNA sequencing were performed by Biomed Co., Ltd. Other chemicals for fluorescence assay, gel electrophoresis, and HPLC analysis were products of Sigma.

### Construction of recombinants

To develop CRISPRi system in *K*. *pneunomiae*, enhanced green fluorescent protein (EGFP) was used as a reporter. Vector ptac-*egfp* was constructed by insertion of tac-*egfp* expression cassette into pET-28a between *Bgl*II and *Xho*I. The CRISPRi vectors targeting *egfp* gene or lactate-producing enzyme genes were constructed by replacement of the sgRNA sequence in vector plv-dCas9-sgRNA [28] (Additional file 2: Fig. S2, Additional file 3: Table S1). Briefly, two complementary single-stranded target sequences were chemically synthesized and annealed to form a 23 bp double-stranded DNA owning cohesive ends matching the BspQI-digested vector. Subsequent ligation resulted in desired CRISPRi vectors. Vector pdCas9 was achieved by deleting the sgRNA sequence in vector plv-dCas9-sgRNA with *Xma*I/NgoMIV, followed by ligation. To ensure efficient inhibition, two candidate guide RNAs targeting the different regions of EGFP promoter were synthesized and the resultant vectors were designated ptaciT_1_ and ptaciT_2_ (Additional file 3: Table S1). For each lactate-producing enzyme gene, two or three CRISPRi vectors were constructed and named after respective gene (Additional file 4: Table S2). For example, ‘placiM’ indicates the CRISPRi vector targeting methylglyoxal synthase gene *mgsA*; ‘placiD’ refers to CRISPRi vector targeting d-lactate dehydrogenase gene *ldhA*; ‘placiL’ indicates CRISPR vector targeting l-lactate dehydrogenase (EC:1.1.1.27) gene *pmb*; ‘placiA’ represents the CRISPR vector targeting lactaldehyde dehydrogenase *alhA*; while ‘placiMALD’ indicates CRISPRi vector simultaneously targeting above four genes (Additional file 3: Table S1).

### Electro-transformation and screening

The Eppendorf tube containing competent *K*. *pneunomiae* cells was embedded in ice for 30 min and then centrifuged to harvest cells. The cells were extensively rinsed with cold ddH_2_O to remove ions and subsequently mixed with vectors. The mixture was added into a MicroPulser Cuvette for electroporation (0.2 cm, 2.5 kV, time duration > 0.5 ms) based on manufacturer’s instructions. After cultivation in LB medium at 37 °C for 1 h, the cells were plated on LB-agar medium containing chloramphenicol (34 µg/mL), kanamycin (50 µg/mL) or both of them depending on experimental requirements (Additional file 4: Table S2).

### Fluorescence assay of CRISPRi strains targeting lactate-producing enzyme genes

To establish CRISPRi system in *K*. *pneunomiae*, a panel of recombinant *K*. *pneunomiae* strains were constructed with EGFP as a reporter. Briefly, vectors ptac-*egfp*, placiT_1_, placiT_2_ (CRISPRi vectors targeting different region of the promoter sequence of EGFP) (Fig. 2), and the control vector plv-dCas9-sgRNA with non-targeting sgRNA, were individually transformed into component *K*. *pneunomiae* cells, resulting in recombinant strains *K*. *pneunomiae*(ptac-*egfp*), *K*. *pneunomiae*(ptac-placiT_1_), *K*. *pneunomiae*(ptac-placiT_2_) and *K*. *pneunomiae*(plv-dCas9-sgRNA), respectively. These recombinant strains were grown in LB medium for 15–20 h, and then transferred to 250 m: flasks containing 100 mL fermentation medium, 85 µg/mL chloramphenicol and 50 µg/mL kanamycin. The strains were cultivated in a rotary shaker at 200 rpm and 37 °C. After 3 h cultivation, IPTG and aTc, at final concentrations of 0.5 mM and 2 µM, respectively, were added into fermentation broth, and the cultivation conditions were adjusted to 30 °C and 150 rpm to induce dCas9 expression. At 18 h, fermentation broth was diluted tenfold to examine OD_600_ using a visible spectrophotometer (APL instrument, Shanghai). The fermentation broth was directly used for fluorescence assay by a fluorescence spectrophotometer (HITACHI).

### Real-time PCR analysis of CRISPRi strains targeting lactate-producing enzyme genes

*Klebsiella pneumoniae* cells were harvested by centrifugation at 12,000 rpm and 4 °C and immediately chilled with liquid nitrogen to avoid RNA degradation. The cells were used for extracting total RNA. For CRISPRi strains Kp(ptac-*puuC *+ placiL), Kp(ptac-*puuC *+ placiD), Kp(ptac-*puuC *+ placiA), Kp(ptac-*puuC *+ placiM) and Kp(ptac-*puuC *+ placiMALD), total RNA was extracted using the RNA prep pure Cell/Bacteria Kit (Tiangen, Beijing, China). The cDNA was synthesized using HiFi-MMLV cDNA Synthesis Kit (CWbio Co. Ltd). The chemically synthesized cDNA was mixed and subjected to gradient dilution and served as template to determine the specificity and efficiency of the primers. qRT-PCR was carried out using UltraSYBR mixture (with ROX) (CWbio. Co. Ltd). The cDNA from each sample was diluted to determine the linear range for qRT-PCR (Fig. 3). 16S rRNA was recruited as the internal standard in qRT-PCR analysis. The statistics was analyzed using 2^−∆∆Ct^ strategy.

### Shake-flask cultivation of CRISPRi strains targeting lactate-producing enzyme genes

The CRISPRi vectors targeting one or four lactate-producing enzyme genes were transformed into *K*. *pneumoniae*(ptac-*puuC*), generating recombinant strains Kp(ptac-*puuC *+ placiM), Kp(ptac-*puuC *+ placiA), Kp(ptac-*puuC *+ placiL), Kp(ptac-*puuC *+ placiD) and Kp(ptac-*puuC *+ placiMALD). The strain harboring vector plv-dCas9 was used as a control (Additional file 3: Table S1). All above recombinant *K*. *pneumoniae* strains were grown in LB medium for 10 h and subsequently transferred to shake flasks containing fermentation medium, 85 µg/mL chloramphenicol and 50 µg/mL kanamycin. These strains were cultivated at 37 °C and 150 rpm. After 3 h cultivation, IPTG and aTc at final concentrations of 0.5 mM and 2 µM, respectively, were added to induce dCas9 expression. The fermentation broth was sampled every 3 h to examine cell growth, glycerol consumption and metabolites formation.

### Bioreactor cultivation of CRISPRi strains targeting lactate-producing enzyme genes

Strains Kp(ptac-*puuC *+ placiD) and Kp(ptac-*puuC *+ placiMALD) were grown in shake-flasks containing LB medium at 37 °C and shaken at 150 rpm. After 24 h cultivation, strains were transferred to 5 L bioreactor (Baoxing, China) containing antibiotics, IPTG and fermentation medium aforementioned. Fermentation conditions were similar to previously reported [2]. Briefly, agitation speed was 400 rpm, air was supplied at 1.5 vvm, pH was maintained at 7.0 by titration with 5 M NaOH. The initial glycerol concentration was 40 g/L. Dissolved oxygen was monitored with electrode. Fermentation broth was sampled every 3 h to examine biomass, residual glycerol and metabolites. Glycerol was supplemented when its concentration dropped below 10 g/L.

### Analytical methods

Cell concentrations were measured by using microplate reader (Multiskan FC, Thermo) at 600 nm with 200 µL fermentation broth added in a cuvette. To measure metabolites, fermentation broth was centrifuged at 12,000 rpm for 10 min to remove bacteria. The 3-HP, lactic acid and acetic acid in supernatant were analyzed by HPLC (Shimazu, Tokyo, Japan) system equipped with a C_18_ column and a SPD-20A UV detector at 210 nm. The column was maintained at 25 °C. The mobile phase was 0.05% phosphoric acid at a flow rate of 0.8 mL/min. 1,3-PDO and 2,3-BDO were analyzed by GC (Persee). Briefly, the sample were evaporated at 80 °C for 40 min to remove water, and then dissolved in ethanol for GC analysis. Analytical pure of 1,3-PDO and 2,3-BDO were used as standard for quantification.

## Additional files

**Additional file 1: Fig. S1.** Conserved lactic acid pathways in diverse species.

**Additional file 2: Fig. S2.** Schematic diagram of CRISPRi vectors and PuuC expression vector.

**Additional file 3: Table S1.** Strains and vectors used in this study.

**Additional file 4: Table S2.** Primers for engineering CRISPRi vectors.

## Abbreviations

EGFP: enhanced green fluorescent protein
3-HPA: 3-hydroxypropionaldehyde
1,3-PDO: 1,3-propanediol
2,3-BDO: 2,3-butanediol
3-HP: 3-hydroxypropionic acid
CRISPRi: CRISPR interference
ALDH: aldehyde dehydrogenase
qRT-PCR: quantitative real-time PCR
GCR: glycerol conversion ratio
GC: gas chromatography
sgRNA: single guide RNA
NHEJ: non-homologous end joining
aTc: anhydrotetracycline
HPLC: high performance liquid chromatography
