# Supplementary material for: Engineering CRISPR interference system in Klebsiella pneumoniae for attenuating lactic acid synthesis
Source: Microb Cell Fact. 2018 Apr 5;17:56. doi: 10.1186/s12934-018-0903-1 (PMC5887262; doi:10.1186/s12934-018-0903-1)
Supplement: Supplementary file 1 — Additional file 1: Fig. S1. Conserved lactic acid pathways in diverse species. [file 12934_2018_903_MOESM1_ESM.docx]

**Fig. S1 Conserved lactic acid pathways in diverse species.**

Different colors indicate diverse species, including eukaryotes and prokaryotes especially lactic acid bacteria with commercial value. One-directional and bidirectional arrows indicate irreversible and reversible catalytic reactions, respectively. (1) D-lactate dehydrogenase (encoded by *ldhA* gene) or isozymes; (2) L-lactate dehydrogenase (*pmd* gene) in *K*. *pneunomiae* or isozymes; (3) glyoxalase III or isozymes; (4) hydroxyacylglutathione hydrolase or isozymes; (5) Lactaldehyde dehydrogenase (*aldA* gene) or isozymes. GADP, glyceraldhyde 3-phosphate; DHAP, dihydroxyacetone phosphate; PEP, phosphoenolpyruvate.
