# Supplementary material for: Engineering CRISPR interference system in Klebsiella pneumoniae for attenuating lactic acid synthesis
Source: Microb Cell Fact. 2018 Apr 5;17:56. doi: 10.1186/s12934-018-0903-1 (PMC5887262; doi:10.1186/s12934-018-0903-1)
Supplement: Supplementary file 2 — Additional file 2: Fig. S2. Schematic diagram of CRISPRi vectors and PuuC expression vector. [file 12934_2018_903_MOESM2_ESM.docx]

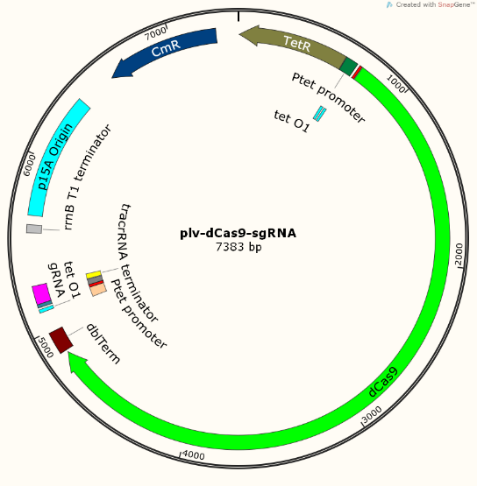

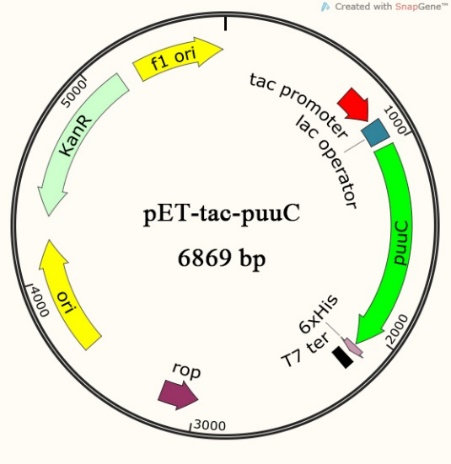


Double digestion with

XmaI/ NgoMIV L → vector pdCas9

Digestion with BspQI→CRISPRi vectors targeting lactate-producing enzyme genes


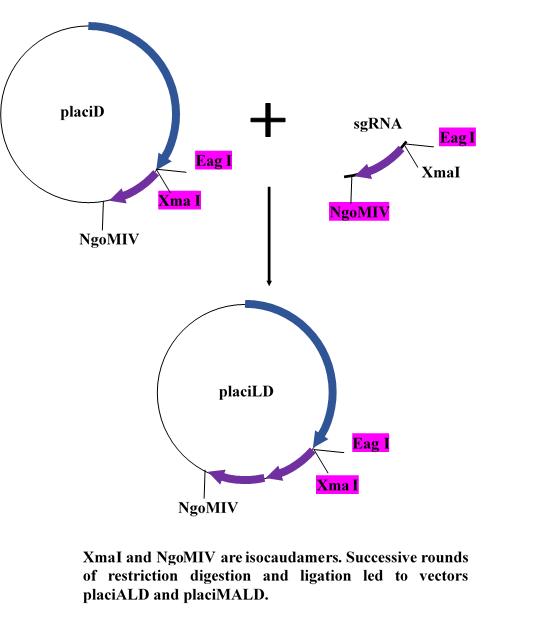


**Fig. S2**. **Schematic diagram of CRISPRi vectors and PuuC expression vector.**

Vector plv-dCas9 was derived from vector plv-dCas9-sgRNA (see Reference 28- Lv L, Ren YL, Chen JC, et al. Metab Eng. 2015; 29, 160–168) by deletion of sgRNA sequence with *Xma* I/NgoM IV, followed by ligation.

The CRISPRi vectors targeting *egfp* gene or lactate-producing enzyme genes were constructed by replacement of the sgRNA sequence in vector plv-dCas9-sgRNA. Briefly, two complementary single-stranded target sequences were chemically synthesized and annealed to form a 23 bp double-stranded DNA owning cohesive ends matching the BspQI-digested vector. Subsequent ligation resulted in desired CRISPRi vectors.

Aldehyde dehydrogenase PuuC expression vector pET-tac-puuC was constructed in our lab (see Reference 2- Li Y, Wang X, Ge XZ, Tian PF. High production of 3-hydroxypropionic acid in *Klebsiella pneumoniae* by systematic optimization of glycerol metabolism. Sci Rep. 2016; 6, 26932).
