# Supplementary material for: Engineering CRISPR interference system in Klebsiella pneumoniae for attenuating lactic acid synthesis
Source: Microb Cell Fact. 2018 Apr 5;17:56. doi: 10.1186/s12934-018-0903-1 (PMC5887262; doi:10.1186/s12934-018-0903-1)
Supplement: Supplementary file 3 — Additional file 3: Table S1. Strains and vectors used in this study. [file 12934_2018_903_MOESM3_ESM.docx]

**Table S1 Strains and vectors used in this study**

| Strains/vectors | Description | Reference/source |
| --- | --- | --- |
| *Klebsiella pneumoniae* DSM 2026 | Wild type | DSMZ |
| *E. coli* Top10 | Competent cells | Biomed |
| pET-28a | Prokaryotic expression vector | Novagen |
| pSg1 | sgRNA vector targeting pBR322 ori, Kan^R^ | This study |
| pSg2 | sgRNA vector targeting pBR322 ori, Kan^R^ | This study |
| ptac-*puuC* | *puuC* expression vector, *tac* promoter, Kan^R^ | (Li Y et al., 2016 ) |
| ptac-*egfp* | EGFP expression vector, *tac* promoter, Kan^R^ | This study |
| plv-dCas9-sgRNA | dCas9 gene and sgRNA under *tet* promoter, Cm^R^ | (Lv et al., 2015) |
| pdCas9 | dCas9 expression vector, Cm^R^ | This study |
| placiT_1_ | CRISPRi vector, targeting *lac* promoter of EGFP, Cm^R^ | This study |
| placiT_2_ | CRISPRi vector, targeting *lac* promoter of EGFP, Cm^R^ | This study |
| placiL_1_ | CRISPRi vector targeting *pmd* gene, Cm^R^ | This study |
| placiL_2_ | CRISPRi vector targeting *pmd* gene, Cm^R^ | This study |
| placiL_3_ | CRISPRi vector targeting *pmd* gene, Cm^R^ | This study |
| placiD_1_ | CRISPRi vector targeting *ldhA* gene, Cm^R^ | This study |
| placiD_2_ | CRISPRi vector targeting *ldhA* gene, Cm^R^ | This study |
| placiA_1_ | CRISPRi vector targeting *aldA* gene, Cm^R^ | This study |
| placiA_2_ | CRISPRi vector targeting *aldA* gene, Cm^R^ | This study |
| placiM_1_ | CRISPRi vector targeting *mgsA* gene, Cm^R^ | This study |
| placiM_2_ | CRISPRi vector targeting *mgsA* gene, Cm^R^ | This study |
| placiMALD | CRISPRi vector simultaneously targeting *mgsA*, *aldA*, *pmd* and *ldhA* genes, Cm^R^ | This study |
| Kp(ptac-*egfp*+ptaciT_1_) | *K*. *pneumoniae* harboring vectors ptac-*egfp* and ptaciT_1_ | This study |
| Kp(ptac-*egfp*+ptaciT_2_) | *K*. *pneumoniae* harboring vectors ptac-*egfp* and ptaciT_2_ | This study |
| Kp(ptac-*egfp* +plv-dCas9-sgRNA) | *K*. *pneumoniae* harboring vectors ptac-*egfp* and plv-dCas9-sgRNA | This study |
| Kp(ptac-*puuC*+placiL_1_) | *K*. *pneumoniae* harboring vectors ptac-*puuC* and placiL_1_ | This study |
| Kp(ptac-*puuC*+placiL_2_) | *K*. *pneumoniae* harboring vectors ptac-*puuC* and placiL_2_ | This study |
| Kp(ptac-*puuC*+placiL_3_) | *K*. *pneumoniae* harboring vectors ptac-*puuC* and placiL_3_ | This study |
| Kp(ptac-*puuC*+placiD_1_) | *K*. *pneumoniae* harboring vectors ptac-*puuC* and placiD_1_ | This study |
| Kp(ptac-*puuC*+placiD_2_) | *K*. *pneumoniae* harboring vectors ptac-*puuC* and placiD_2_ | This study |
| Kp(ptac-*puuC*+placiA_1_) | *K*. *pneumoniae* harboring vectors ptac-*puuC* and placiA_1_ | This study |
| Kp(ptac-*puuC*+placiA_2_) | *K*. *pneumoniae* harboring vectors ptac-*puuC* and placiA_2_ | This study |
| Kp(ptac-*puuC*+placiM_1_) | *K*. *pneumoniae* harboring vectors ptac-*puuC* and placiM_1_ | This study |
| Kp(ptac-*puuC*+placiM_2_) | *K*. *pneumoniae* harboring vectors ptac-*puuC* and placiM_2_ | This study |
| Kp(ptac-*puuC*+placiMALD) | *K*. *pneumoniae* harboring vectors ptac-*puuC* and placiMALD | This study |
| Kp(ptac-*puuC*+pdCas9) | *K*. *pneumoniae* harboring vectors ptac-*puuC* and pdCas9 | This study |
