# Supplementary material for: Engineering CRISPR interference system in Klebsiella pneumoniae for attenuating lactic acid synthesis
Source: Microb Cell Fact. 2018 Apr 5;17:56. doi: 10.1186/s12934-018-0903-1 (PMC5887262; doi:10.1186/s12934-018-0903-1)
Supplement: Supplementary file 4 — Additional file 4: Table S2. Primers for engineering CRISPRi vectors. [file 12934_2018_903_MOESM4_ESM.docx]

**Table S2 The sgRNA primer used in this study**

| **Primer** | **Sequence** | **Vector** |
| --- | --- | --- |
| Lsg1-F | AAAttcagagggttagcgcaataggc | placiL1 |
| Lsg1-R | AACgcctattgcgctaaccctctgaa |  |
| Lsg2-F | AAAcatattcagatctcaagtacgaa | placiL2 |
| Lsg2-R | AACttcgtacttgagatctgaatatg |  |
| Lsg3-F | AAActcgcaaatgctctggttgagca | placiL3 |
| Lsg3-R | AACtgctcaaccagagcatttgcgag |  |
| Dsg1-F | AAAtttctccagtgattataccgtca | placiD1 |
| Dsg1-R | AACtgacggtataatcactggagaaa |  |
| Dsg2-F | AAAttctagagcatatcgcggacttg | placiD2 |
| Dsg2-R | AACcaagtccgcgatatgctctagaa |  |
| Asg1-F | AAAcgggtgttgaacgggtgctgtca | placiA1 |
| Asg1-R | AACtgacagcacccgttcaacacccg |  |
| Asg2-F | AAAacgaactggccatcaatatacat | placiA2 |
| Asg2-R | AACatgtatattgatggccagttcgt |  |
| Msg1-F | AAAacctgcaaacagattactgaatg | placiM1 |
| Msg1-R | AACcattcagtaatctgtttgcaggt |  |
| Msg2-F | AAAggagacgagattaccggtagtgc | placiM2 |
| Msg2-R | AACgcactaccggtaatctcgtctcc |  |

F, forward; R, reverse.

placiL1, placiL2 and placiL3 indicate three candidate CRISPRi vectors targeting lactate-producing enzyme gene *pmd*.

placiD1 and placiD2 indicate two candidate CRISPRi vectors targeting lactate-producing enzyme gene *ldhA*.

placiA1 and placiA2 indicate two candidate CRISPRi vectors targeting lactate-producing enzyme gene *alhA*.

placiM1 and placiM2 indicate two candidate CRISPRi vectors targeting lactate-producing enzyme gene *mgsA*.
